# The unique resistance and resilience of the Nigerian West African Dwarf goat to gastrointestinal nematode infections

**DOI:** 10.1186/1756-3305-4-12

**Published:** 2011-02-03

**Authors:** Samuel N Chiejina, Jerzy M Behnke

**Affiliations:** 1 Faculty of Veterinary Medicine, University of Nigeria, Nsukka Nigeria; 2 School of Biology University of Nottingham, University Park, Nottingham NG7 2RD, UK; 3 College of Veterinary Medicine, Michael Okpara University of Agriculture, Umudike, Abia State, Nigeria

## Abstract

**Background:**

West African Dwarf (WAD) goats serve an important role in the rural village economy of West Africa, especially among small-holder livestock owners. They have been shown to be trypanotolerant and to resist infections with *Haemonchus contortus *more effectively than any other known breed of goat.

**Methods:**

In this paper we review what is known about the origins of this goat breed, explain its economic importance in rural West Africa and review the current status of our knowledge about its ability to resist parasitic infections.

**Conclusions:**

We suggest that its unique capacity to show both trypanotolerance and resistance to gastrointestinal (GI) nematode infections is immunologically based and genetically endowed, and that knowledge of the underlying genes could be exploited to improve the capacity of more productive wool and milk producing, but GI nematode susceptible, breeds of goats to resist infection, without recourse to anthelmintics. Either conventional breeding allowing introgression of resistance alleles into susceptible breeds, or transgenesis could be exploited for this purpose. Appropriate legal protection of the resistance alleles of WAD goats might provide a much needed source of revenue for the countries in West Africa where the WAD goats exist and where currently living standards among rural populations are among the lowest in the world.

## Background

**Figure 1:**
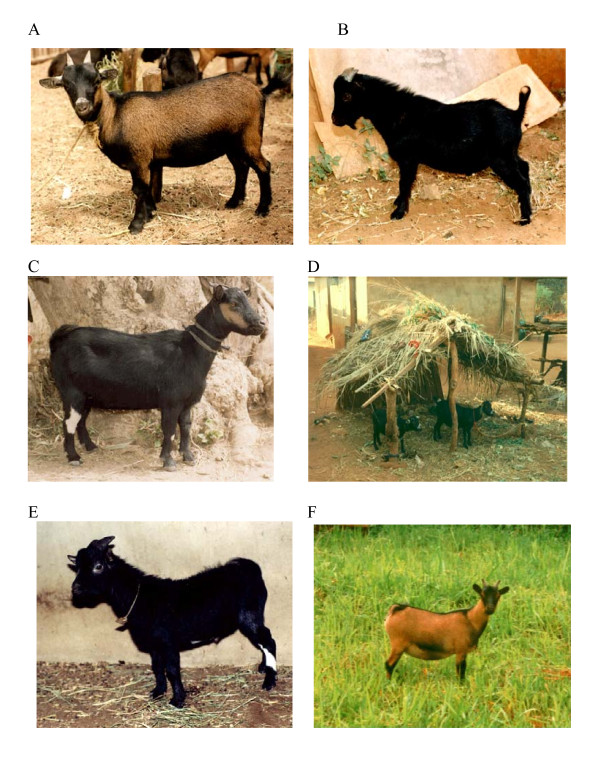
**Examples of Nigerian WAD goats in various settings**. A, Savanna zone WAD goat doe from northern Nigeria. B, Savanna zone WAD goat buck from northern Nigeria. C, Savanna zone WAD goat doe, to illustrate a contrasting colour morph to those shown in A and B above. D, Two Nigerian humid zone WAD goats in a typical village setting. E, Adult, breeding humid zone WAD goat buck in the animal house at the University of Nigeria Nsukka. F, Humid zone WAD goat doe in the external paddock of the animal house at the University of Nigeria Nsukka, showing one of the common colour morphs.

The major contributor of the modern domestic goat, *Capra hircus*, is believed to be the wild Bezoar goat, *Capra aegagrus *distributed from the mountains of Asia Minor [1], across the Middle East. There are ten primary goat breeds to which other modern breeds worldwide are traceable, namely Alpine, Angora, Boer, Cashmere, Le Mancha, Nubian, Oberhasli, Pigmy, Saanen and Toggenburg. The present day dwarf goats of West and Central Africa correspond to the so-called pigmy goat but the recognised name for the breed in the region is the West African Dwarf (WAD) goat (Figure 1). However, other names such as Cameroonian, Nigerian, Guinean and Fouta Djallon are sometimes used to describe WAD goats found in particular countries in the region. These may be considered as varieties or ecological types (ecotypes) of WAD goat, which have adapted to the different ecosystems in the region. They are found, predominantly in the humid and sub-humid, and also in the drier, savanna climates, below latitude 14° north. One popular belief, based on few documented facts, is that all dwarf goats found in West and Central Africa, England, Sweden, Germany and North America originated from the Cameroonian Dwarf goat [2], although, based on their morphology in relation to other dwarf goat breeds, it has been suggested that the Nigerian WAD goats may have a different, but as yet unknown, origin [3,4]. However, genetic and archaeological evidence of the precise origins of WAD goats are still lacking.

Nevertheless, recent work has shown that Nigerian WAD goats are endowed with the capacity to resist trypanosome and intestinal nematode infections more effectively than any other known breed of goat. Since there is no easily accessible, systematic review of published information on controlled experimental GI nematode infections of these animals, in this article we first explain the importance of WAD goats to the local livestock owning communities of West Africa, then review the available information on their capacity to resist parasitic infections, suggest possible explanations for these unique traits and indicate how they may be exploited in the broader context to improve the resilience of goats, and hence their health and productivity worldwide.

### The importance of the WAD goat industry in West Africa

Goats account for about 30% of Africa's ruminant livestock and produce about 17% and 12% of its meat and milk respectively [5]. Sub-Saharan Africa (SSA) accounts for over 60% of the total goat population in Africa, with an estimated 147 million goats representing about 80 indigenous breeds distributed across all agro-ecological zones and ruminant livestock production systems [6]. The WAD goat is the commonest and most important indigenous goat breed in the 18 countries of West and Central Africa [7], most of which (except Mali, Burkina Faso and the Central African Republic) have an Atlantic coastline namely, Senegal, The Gambia, Guinea Bissau, Guinea, Sierra Leone, Liberia, Mali, Upper Volta, Cote d'Ivoire, Ghana, Togo, Benin, Nigeria, Cameroon, Central African Republic, Gabon Congo and Zaire) [8]. Nigeria hosts the largest WAD goat population with approximately 11 million in the humid zone of Eastern Nigeria. There are two major ecotypes: the humid zone and the savanna WAD goats, (Figure 1) and these differ in several respects, notably their body weight, the latter being about 2-3 kg heavier on average at 12 months of age [9]. It is estimated that at least 90% of these animals are owned by small-holder rural goat keepers, for whom goats represent an important asset [10].

Women and children play a pivotal role in WAD goat husbandry. Children normally herd goats, while their day-to-day management and the care of young stock usually fall to women. They are generally kept in small herds on mixed farms and provide their owners with a broad range of products and socio-economic services such as cash income (meat), security (milk), gifts (skin), and manure for the crops. Left-overs from the domestic kitchen, which are provided by the womenfolk, and cut-and-carry fodder/foliage, which are the responsibility of children and the men folk, are important ingredients in the husbandry of goats in rural areas. Therefore, goats not only play a vital role in ensuring food security of a household (often being the only asset possessed by a poor household), but when needed and in time of trouble (e.g. crop failure or family illness, school fees) goats may be sold to provide an important source of cash [11]. Any intervention aiming to improve goat productivity will therefore have an immediate socio-economic impact on rural communities, especially the poorest of these for whom goats represent the only livestock they can afford to raise. The socio-economic importance of WAD goats in the area is best illustrated by the terms: 'cow of the poor' and 'bank on the hoof', which are commonly used to describe them.

Important attributes of the WAD goat include its excellent adaptation to its native habitat, high fertility, and prolificacy. However, their most important attributes are their resistance to the important insect-borne disease, trypanosomosis (trypanotolerance), and to GI nematodes (see below). These attributes have enabled the predominantly small-scale rural goat keepers in the area to successfully rear, and continue to derive their sustenance from these animals without recourse to the use of trypanocides and anthelmintics, which are neither affordable nor available to most of them. Other breeds do not survive long in the humid zones of Nigeria, because they succumb rapidly to trypanosomosis.

### **The gastrointestinal (GI) nematodes of indigenous WAD goats of West Africa**

Available records from field surveys and other epidemiological data indicate that WAD goats from all the countries of the region, such as Ghana [12], Mali [13], Nigeria [14] and Sierra Leone [15], are parasitized by essentially the same genera and species of GI nematodes namely: *Haemonchus contortus *and *Trichostrongylus axei *in the abomasum, *T. colubriformis, Bunostomum trigonocephalum, Gaigeria pachyscelis, Cooperia curticei, C. punctata*, and *Strongyloides papillosus *in the small intestine and *Oesophagostomum columbianum, Trichuris ovis, T. globulosa *and *Chabertia ovina *in the large intestine. However, the commonest and most important are *H. contortus*, *T. colubriformis *and *O. columbianum *[16]. Under the predominantly traditional, small-scale system of goat husbandry and ownership in the region, in which little or no formal worm control is practiced, low level chronic infections occur all the year round, with prevalence of infections of 80 - 100 percent at the peak of the rainy season [16,17]. Such widespread, insidious, chronic infections are considered to be a major contributory factor to poor productivity of WAD goats in many countries in the area [12, 18-20). However, a few cases of clinical parasitic gastroenteritis (PGE), with high mortalities have been described in intensively grazed goats [21]. In such situations, intensive grazing, absence of formal worm control measures, coupled with poor hygiene, give rise to heavy infections in kids, sometimes complicated by other factors notably malnutrition and concurrent infections with coccidiosis, ticks, lice, viruses and extensive mange mite infestations [21].

### WAD goat - GI nematode interactions in experimental and naturally acquired infections

A good deal of our knowledge about GI nematode infections in WAD goats is derived primarily from clinical case records and epidemiological data from field surveys. Controlled laboratory studies on host-parasite interactions have been lacking, until recently. Therefore, it has been assumed that WAD goats, like other goat breeds world-wide, are more susceptible to these nematodes than sheep, and are less able to control the infections and the associated pathophysiological consequences, as a result of poorly developed and less effective immune responses to them [22]. The need for more studies of goat-GI nematode interactions was stressed by Hoste et al [22], particularly in relation to the increasing realisation that there are indeed important differences between sheep and goats in their capacity to regulate their GI nematode infections [23-26]. Thus, of the four distinctive manifestations of host acquired resistance to GI nematodes in sheep, namely poor establishment of infective larvae (L3), reduced worm development and growth, reduced worm fecundity and accelerated/rapid worm rejection, only the last two are believed to be expressed by goat breeds.

It has also been suggested that the ability of goats to control challenge infections following a primary infection is weaker than that of sheep and that immunological memory following anthelmintic abbreviation of a primary infection and challenge does not last as long as in sheep [27]. Furthermore, although goats show evidence of immune regulation of GI nematodes, it is believed that they do not develop full immunological responsiveness until 12 months of age compared with 6 months for sheep [21,28]. Importantly, WAD goats are different to other breeds in this respect, as shown by Ayeni [29] who found that WAD goat kids were able to mount strong immune responses to chicken red blood cells, comparable to adult goats, from three months of age. This suggests that WAD goats, which are usually fully sexually mature at 6-7 months of age [30], attain immunological maturity much earlier than most other goat breeds. In most caprine studies little attention has been given to possible differences between and within goat genotypes from different geo-climatic zones of the world in their responses to their core parasites and yet different breeds have adapted to radically different ecosystems, goat husbandry, production systems and parasite strains typically encountered under local conditions. It is unlikely that all breeds, as also all individuals within specific breeds, will respond identically to GI nematodes, nor indeed to any other infectious organisms. Some diversity in response to their native strains of parasites would be expected.

We have conducted a series of controlled experiments on the parasitological and clinico-pathological responses of two ecotypes of WAD goats found in the southern humid and northern savanna zones of Nigeria to their native strains of *H. contortus *[9,31-33]. These studies were later extended to include naturally acquired GI nematode infections in these contrasting geo-climatic zones [34,35]. Our data do not support the generally held view about the high susceptibility of goats to GI nematodes and especially to *H. contortus*, and their inability to control the pathophysiological consequencies of these infections. On the contrary, both WAD goat ecotypes appear to be naturally endowed with unusually strong resistance and resilience to their native strains of GI nematodes but particularly *H. contortus *the only species to have been studied experimentally thus far [9,32,33].

Our laboratory studies employed a variety of infection protocols in 7-9 month old kids, which included single pulse infections ranging from 3000 to 6000 L3 [9,32], trickle and rapidly escalating, immunising infections, followed eight weeks later by chemical abbreviation of infections and, in some animals, challenge with 4000-6000 L3 [33,36]. The results consistently showed extremely low worm establishment and recovery during the prepatent and patent phases of infections, 14-18 and 18-110 days post infection (pi), respectively. In one typical study [9] approximately 83% of goats harboured less than 1% of the administered dose of 6000 L3 18 days pi. The majority of goats had no worms at all while a few, susceptible individuals, carried 500-1070 worms. It is not surprising that these low level infections were not associated with clinical manifestations or measurable losses in production. Moreover, truncation of an immunising infection with Fenbendazole, markedly boosted resistance to challenge, thereby resulting in almost total elimination of the challenge dose 14 days pi [33]. This is indicative of effective immunological memory, which is said to be either lacking or poorly expressed by other breeds of goats, although we have not carried out any studies to ascertain how long this memory might last. Our data also suggest that immune responsiveness is fully expressed by 7-9 month old Nigerian WAD goats. This degree of resistance and resilience to *H. contortus *has not been reported for any other breed of goat, including WAD goats from other parts of West Africa, where no specific, controlled studies on GI nematode-WAD goat interactions appear to have been carried out. All the laboratory studies on GI nematode infections in WAD goats in the Gambia [37] involved concurrent infections with *Trypanosoma congolense *and so did not specifically address GI nematodes alone. Nevertheless, the limited available data suggest that WAD goats in that part of West Africa are highly susceptible to *H. contortus *and other GI nematodes.

**Figure 2:**
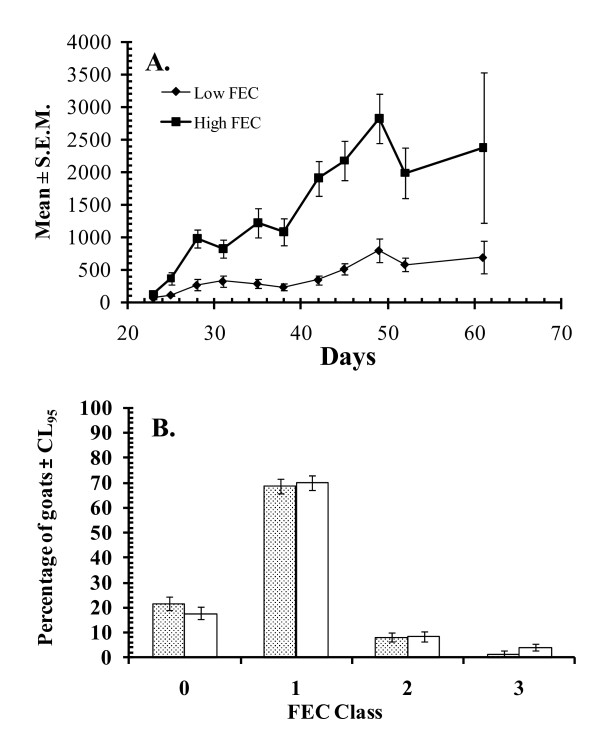
**Strong responder (low FEC) and poor responder (high FEC) phenotypes among Nigerian WAD goats**.* A. Haemonchus contortus *faecal egg counts in two groups of humid zone Nigerian WAD goats. All the animals were given the same exposure to infective larvae of the parasite: 500 L3 on day 0, 1000 on d9 and d16, 2000 on d 23 and 32 and 3000 on d39. The High FEC values are the mean (± S.E.M.) FEC from the 18 goats with highest overall total egg output over the period of observation and the Low FEC group are the 18 goats with the lowest values across the period. Three goats with intermediate FEC were not included in these calculations. For further details see ref [36]. B. Distribution of FEC phenotypes in naturally acquired infections. Overall percentage of high, intermediate and low infection levels with GI nematodes in savannah WAD goats, based on faecal egg counts (FEC), as reflected in the percentage of goats at two markets (Akpagher and Gboko), classified in FEC class 0 (no eggs detected), FEC class 1 (1-50EPG), FEC class 2 (51-1500 EPG) and FEC class 3 (>1500 EPG). Akpagher is shown in stippled columns and Gboko in open columns. The predominance of low FEC (strong responder) phenotypes is apparent. For further details see ref [35].

The other characteristic feature of Nigerian WAD goat-GI nematode interactions demonstrated in naturally acquired infections was the striking individual variability in faecal egg counts (FEC) and worm burdens, which allowed identification and segregation of goats from both the humid and savanna zones into strong and relatively weak responder, FEC phenotypes (Figure 2). The former phenotype, with FECs of only 0-50 eggs per gramme (epg) of faeces under field conditions, constituted approximately 76-80 and 80-85% respectively, of the population of all goats examined during the rainy season in the two zones (Figure 2B) when goats are usually exposed to the highest levels of infection [38]. Broadly similar variability in FEC between and within breeds has been reported in sheep [39-41] and goats [42-44] from different parts of the world and so is not peculiar to Nigerian WAD goats. What is unique about the latter is the exceptionally strong degree of resistance demonstrated, particularly in *H. contortus *infections, and the preponderance of the resistant phenotype in WAD goat populations from the southern humid to the northern savanna zones of the country. This was the basis for the use of the term haemonchotolerance [32] to describe this phenomenon with reference to *H. contortus*. We believe that haemonchotolerance is an innate characteristic of Nigerian ecotypes of WAD goat.

### The basis of the unique resistance and resilience of Nigerian goats to GI nematodes

It is not clear why the Nigerian WAD goat, in contrast to goats in other countries of West Africa and possibly elsewhere, are capable of effective control/regulation of *H. contortus *infections both in laboratory and naturally acquired infections. Although there are a number of plausible explanations, which have yet to be examined experimentally, we believe that our data are consistent with the view that the phenomenon is essentially genetically determined and expressed via, as yet undetermined, host immunological responses [31]. The other possible, contributory factor includes low infectivity and virulence/pathogenicity of the humid and savanna zone isolates of these parasites.

### Host genetics

The genetic basis of GI nematode resistance in sheep and goats and the value of FEC as a phenotypic marker and selection criterion for the trait are well known and well documented [44-47]. We hypothesise that nature and nurture have interacted to produce a goat genotype in Nigeria, the Nigerian WAD goat, with unique GI nematode resistance and resilience, which would have taken decades of expensive research to produce. However, the intriguing question is why WAD goats in other countries of West Africa do not appear to possess the same trait. A recent genetic study may provide a clue. Fidalis [48] has shown, using molecular genetics tools, that WAD goats in many countries of the region are no longer pure-bred as a result of introgression of susceptibility alleles of nematode resistance genes from Sahelian goats. Furthermore this phenomenon has spread from North Senegal down to Guinea and eastwards to Mali. His study did not extend to more southern coastal West African countries such as Nigeria and Ghana with large populations of humid zone WAD goats.

**Figure 3:**
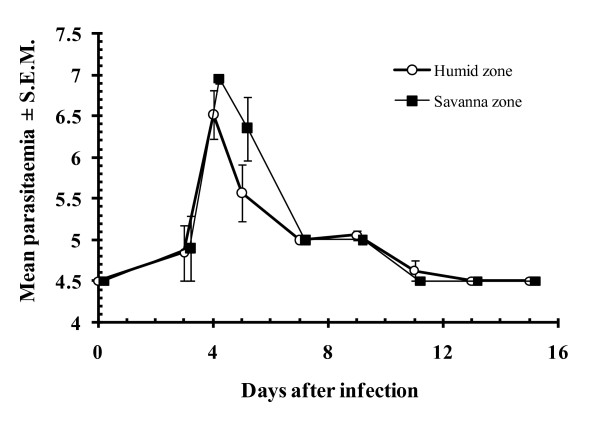
**The course of parasitaemia with Trypanosoma brucei in experimentally infected humid zone and savannah zone WAD goats**. The goats were infected on day 0 with 5 × 10^6 ^organisms, in 100 μl of blood by subcutaneous inoculation. Mean parasitaemia is given in log_10 _units per ml of blood and 4.5 on the y-axis represents the limit for detection. The figure shows that both the humid and the savanna zone WAD goats sustained a limited infection with parasitaemia dropping below the levels of detection in the second week after infection. For further details see ref [9].

Corroborative evidence for the genetic findings was provided by studies on trypanotolerant WAD sheep and goats in the Gambia [reviewed in [49]], which showed that the well known trypanotolerant dwarf breeds of sheep, the Djallonke, and WAD goats in the areas studied by Fidalis [48] have lost a significant degree of their resistance to trypanosomes thereby making them as susceptible to trypanosomes as trypanosusceptible Sahelian breeds [37,50]. Loss of trypanotolerance was particularly evident in concurrent infections with *H. contortus*. If Sahelian gene introgression was responsible for the abrogation or attenuation of this genetically determined survival trait in WAD goats in those countries it is also likely to have done the same to GI nematode resistance and hence the relatively high susceptibility of their WAD goats to GI nematodes. Although we have no molecular genetic information supporting the idea that Nigerian WAD goats are pure, without extensive introgression from other breeds that might weaken their natural trypanotolerance (Figure 3) and haemonchotolerance, this is a hypothesis that can be tested easily.

**Figure 4:**
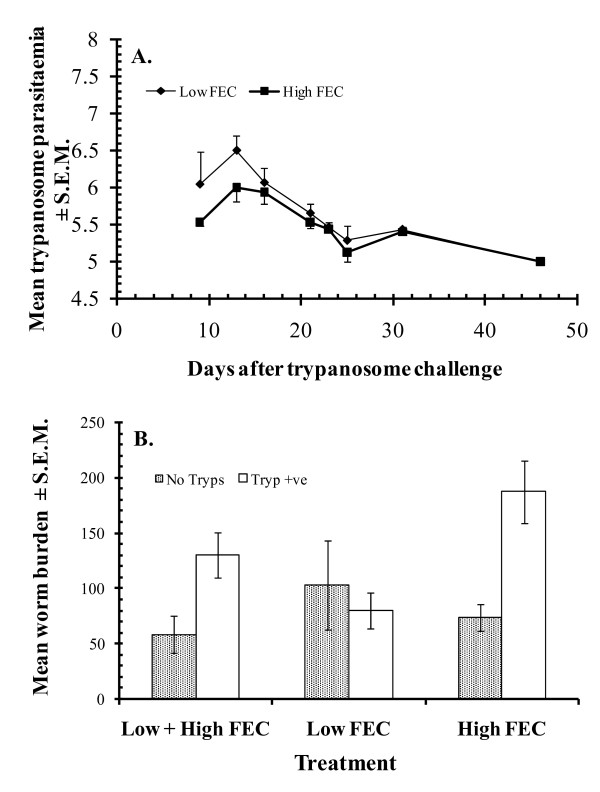
**Concurrent infections with *H. contortus *and *Trypanosoma brucei *in Nigerian WAD goats from the humid zone**. Animals were segregated into low and high FEC producers as described in the legend to Figure 2. All the animals were treated with an anthelmintic on day 61 to remove the worms and then half of each group (9 goats in each) was infected with 50 × 10^6 ^trypanosomes (Tryp +ve, animals infected with *T. brucei*; No Tryps - control groups not infected with *T. brucei*)). Seven days later, on d67 all the animals (*n*=36) were challenged with 3000 L3 of *H. contortus*. The figure shows (A) that the course of the trypanosome challenge was very similar in both groups irrespective of whether they initially showed high or low FEC, and (B) that those animals that harboured heavy infections with worms initially, were cleared of infection, then infected with trypanosomes and challenged with *H. contortus*, developed heavier worm burdens compared with similarly treated animals which produced initially only low intensity FEC. The y-axis indicates the value of the mean worm burden of relevant groups. For further details see ref [36].

We have yet to conduct comprehensive immunological studies on GI nematode infections in WAD goats, although pilot data regarding antibody responses (serum IgG to antigens of *H. contortus*) and eosinophilia are already available [31,51], confirming that some of the arms of Th-2 type responsiveness are activated in GI nematode infected WAD goats. In one study there was clear evidence of more rapid mobilisation of eosinophils with an enhanced eosiniphilia in immunized-challenge groups of WAD goats, although none of a secondary IgG antibody response [51], but the former was not confirmed when the experiment was repeated under slightly different conditions [31]. Although attempts to correlate these responses to worm burdens were less successful, strong immunological basis of resistance can be inferred also from the very efficient control of infections, especially challenge, following anthelmintic abbreviation of immunising infections. Data from concurrent *T. brucei-H. contortus *interactions in our WAD goats [36] are also relevant. Concurrent infections with these two highly pathogenic parasites of ruminants in sub-Saharan Africa, are characterised by a marked increase in FEC, worm burdens and diminished *H. contortus*-specific serum antibody responses, with far reaching pathophysiological consequences, in N'dama cattle [52], and dwarf sheep and goats [37,50] in the Gambia. This is as a result of trypanosome-elicited immunosuppression, resulting in down-regulation of host resistance to the nematode. Crucially, these effects do not occur in Nigerian WAD goats, except for a small but significant increase in the worm burdens of a minority of weak responder phenotypes of goat [Figure 4, [36]]. This is most unusual and suggests that trypanotolerance, which is very strong in Nigerian WAD goats [9], and haemonchotolerance coexist under field conditions in this goat genotype. In a recent field study in a savanna goat population in northern Nigeria [35] we provided evidence in support of this hypothesis. We do not know of any other species of livestock, including dwarf goats in trypanosome-endemic zones of sub-Saharan Africa, which is known to possess and express both of these important survival traits in concurrent infections.

### Parasite strain and virulence

The possibility that the L3 of the GI nematodes we used in our studies were of low infectivity and/or virulence is currently being investigated. Fortunately, an isolate of *H. contortus *from Sahel goats of Northern Nigeria was recently shown to be highly pathogenic for the Sahelian goat breed, the Sokoto Red. A single dose of 5000 L3 produced acute, rapidly fatal haemonchosis in this goat breed [53]. Geographic and host adapted strains/lines of nematodes with genetic and phenotypic differences, and possibly different virulence, undoubtedly exist in nature [54-57]. Therefore, this cannot be ruled out with regard to Nigerian *H. contortus *isolates from the humid and the Sahel zones. However, despite the reported genetic differences between isolates of *H. contortus*, and their phenotypic differences, their effects on the host in terms of pathogenicity and host response, whilst showing some variability, do not differ markedly between isolates. Therefore, it is unlikely that the strain of nematodes we studied significantly influenced our WAD goat data, particularly with regard to the existence of two contrasting response phenotypes of goat, one of which, the predominant phenotype, is strongly resistant and the other fully susceptible to infection, and the effect of trypanosome-elicited immunosuppression, which targets and modulates only the worm burdens of the weak responder phenotypes. This is also a testable hypothesis.

### Browsing versus grazing

Hoste et al [25] have discussed the influence of browsing and grazing behavioural responses of goats and sheep respectively on the evolution of caprine and ovine immune responses to GI nematode infections. The browsing behaviour of goats, which limits contact with nematode L3 in the environment, especially at soil level, is believed to have contributed to the evolution of less effective immunoregulatory mechanisms in this host than in sheep. Therefore, goats should not normally be expected to carry heavy GI nematode infections in their natural environments, except when confined in, or are grazed on, heavily contaminated pasture, without choice of browse [21]. In such artificial/unnatural scenarios goats acquire heavier infections than sheep [23,26]. We cannot identify any behavioural responses or managerial/husbandry practices, which could have accounted for the unusually strong resistance of Nigerian WAD goats to their native strain of GI nematodes. All indications are that the prevailing traditional small-scale husbandry systems of goat management in Nigeria [14] should ensure exposure to generally low to moderate levels of infection, which based on work in other breeds (see above) in turn should result in weakly developed immunoregulatory mechanisms in the goats. The latter was clearly not the case.

### Concluding remarks

The Nigerian WAD goat-GI nematode model has revealed departures from a number of stereotypical views about the immunological responses of goats to GI nematodes namely:

• Immune responses which limit/control establishment of L3, worm development and growth are lacking in goats. We recorded as low as 0-1% worm recovery 14-18 days after a single primary infection.

• Immunological memory required to control challenge infections following anthelmintic abbreviation of a primary infection is weak and short-lived in goats. In our laboratory infection studies truncation of immunising infections boosted resistance (evidenced by worm rejection) of WAD goats to challenge infection but at this stage we do not know how long this memory might last.

• The capacity to respond immunologically to GI nematodes does not develop in goats before 12 months of age. We do not have direct evidence to the contrary, except that all the goats which we used in our laboratory studies were aged between 7 and 9 months and most expressed strong resistance and resilience to primary and challenge infections. Furthermore, the experimental studies by Ayeni [29] suggest that three month-old Nigerian WAD goats are capable of responding immunologically to the same degree as adult goats, albeit to chicken RBCs.

We do not know why Nigerian WAD goats appear to be the only reported goat breed that is so strongly resistant to GI nematodes. Why do WAD goats in neighbouring countries at least not express that same responsiveness? We suggest two possible explanations

• Our data are consistent with a genetically determined trait. We believe that this trait is a breed characteristic, particularly with regard to haemonchotolerance, since about 80 % of WAD goats across the country belong to the strong responder phenotype. This trait finds expression via host immune responses in which the majority respond more robustly than the minority, the weak responders (assessed using the four manifestations of acquired resistance to GI nematodes listed above). The reported spread of Sahelian gene introgression into WAD goats in other more northern coastal West African countries might have contributed to their goats being highly susceptible, as they are no longer purebred. The truth is that no controlled studies on WAD goat - *H. contortus *interactions appear to have been conducted outside Nigeria

• Parasite strain and virulence might also have played a part but we believe that their role was secondary. This is testable using the two strains/isolates we have in Nigeria.

• Other factors including peculiarities in behavioural responses, goat husbandry, self medication through consumption of locally available browse plants with anthelmintic properties etc, are remote possibilities. They are possibly relevant under field conditions, but certainly do not apply to our experimental infection studies.

The Nigerian WAD goat - GI nematode interaction is a model worthy of greater attention especially for the elucidation of various aspects of the goat-GI nematode relationship. Examples are the genetic and immunological basis of this relationship. Perhaps the most important implication of our studies, assuming that the trypanotolerant and haemonchotolerant conditions are under relatively simple genetic control, is that it should be possible to exploit the alleles of relevant "resistance" genes to improve disease resistance in the highly susceptible but productive breeds of goats farmed in developed countries for milk and wool. Whether this is possible through conventional breeding, and hence long-term introgression, is not clear, because WAD goats are so much smaller than the much larger productive breeds. However, if the genes involved were to be identified, then transgenesis would open the way for introducing the resistance alleles into productive breeds. This would benefit immensely goat husbandry throughout the world, because GI nematode infections are widely considered to be the major disease causing pathogens of these animals, especially where anthelmintic resistant strains of parasitic nematodes have evolved [47]. Indeed, on farms where triple resistance (resistance to the benzimidazoles, the acetylcholine agonists and the macrocyclic lactones) has evolved [58,59], conventional anthelmintic treatment is no longer effective and environmental control is the only currently available strategy. Thus breed improvement through resistance genes would benefit greatly livestock agriculture in such cases. Moreover, appropriate legal protection of the resistance alleles of WAD goats might provide a much needed source of revenue for the countries in West Africa where the WAD goats exist.

We have attempted to demonstrate in this review that the WAD goats of Nigeria are a unique resource in terms of a hitherto only poorly studied genotype of goat, but which has immense potential to improve goat husbandry throughout the world, based on their phenotypically demonstrable capacity to resist both GI nematode infections and trypanosomes. If global goat husbandry is to benefit from the genetic resource underlying the observed capacity to resist parasitic infections, there is little time to lose, because as has been found in other countries of West Africa [48,49], the Nigerian breed is unlikely to stay pure for much longer. In the pursuit of higher outputs/financial gains farmers are likely soon to introduce larger, more productive and yet more parasite susceptible breeds into the southern regions of Nigeria, and in fact we are aware that this is already beginning to happen in Nigeria. The ultimate prize of a breed of goat that combines high productivity and parasite resistance is worth pursuing, as resistance to chemotherapy spreads globally and intensifies making goat husbandry ever more expensive. Moreover consumer resistance to chemically dosed animals and the increasing appeal of organically farmed meat have generated further pressures on the financial viability of livestock agriculture. Genetically modified (GM) goat breeds, whilst currently subject to yet another source of resistance from the anti GM lobby, might nevertheless in the long-term become accepted and eventually achieve a suitable compromise for both farmers and consumers.

## Competing interests

The authors declare that they have no competing interests.

## Authors' contributions

SNC and JMB wrote the manuscript together, and both have read and approved this final version.

## Acknowledgements

We wish to acknowledge the tremendous support that we have had from the Sir Halley-Stewart Trust, over a period of more than a decade. None of the work described in this review would have been possible without the dedication and enthusiastic contributions from our co-authors over the years. We are much indebted to them all.
